# Selection of Wild Lactic Acid Bacteria Strains as Promoters of Postbiotics in Gluten-Free Sourdoughs

**DOI:** 10.3390/microorganisms8050643

**Published:** 2020-04-28

**Authors:** Bogdan Păcularu-Burada, Luminița Anca Georgescu, Mihaela Aida Vasile, João Miguel Rocha, Gabriela-Elena Bahrim

**Affiliations:** 1 Faculty of Food Science and Engineering, Dunărea de Jos University of Galati, Domneasca Street No. 111, 800201 Galati, Romania; bogdan.pacularu@ugal.ro (B.P.-B.); luminita.georgescu@ugal.ro (L.A.G.); aida.vasile@ugal.ro (M.A.V.); 2 REQUIMTE–Rede de Química e Tecnologia, Laboratório de Química Verde (LAQV), Departamento de Química e Bioquímica, Faculdade de Ciências da Universidade do Porto (FCUP), Rua do Campo Alegre, s/n. P-4169-007 Porto, Portugal; jmfrocha@fc.up.pt

**Keywords:** lactic acid bacteria, newly wild strains, chickpea, quinoa and buckwheat flours, gluten-free sourdough, antimicrobial activity, exopolysaccharides, biotics for life

## Abstract

The occurrence of inflammatory responses in humans is frequently associated with food intolerances and is likely to give rise to irritable bowel disease. The use of conventional or unconventional flours to produce gluten-free baking doughs brings important technological and nutritional challenges, and the use of the sourdough biotechnology has the potential to overcome such limitations. In addition, the typical metabolic transformations carried out by Lactic Acid Bacteria (LAB) can become an important biotechnological process for the nutritional fortification and functionalization of sourdoughs due to the resulting postbiotics. In such a context, this research work aimed at isolating and selecting new LAB strains that resort to a wide range of natural environments and food matrices to be ultimately employed as starter cultures in gluten-free sourdough fermentations. Nineteen LAB strains belonging to the genera of *Lactobacillus*, *Leuconostoc*, *Pediococcus*, and *Streptococcus* were isolated, and the selection criteria encompassed their acidification capacity in fermentations carried out on chickpea, quinoa, and buckwheat flour extracts; the capacity to produce exopolysaccharides (EPS); and the antimicrobial activity against food spoilage molds and bacteria. Moreover, the stability of the LAB metabolites after the fermentation of the gluten-free flour extracts submitted to thermal and acidic treatments was also assessed.

## 1. Introduction

There is an increasing interest of consumers for emerging strategies that are able to promote a healthy lifestyle and the healing of certain diseases through non-invasive methods. In such a circumstance, sourdough bread and other sourdough-based baking goods (e.g., biscuits, crackers, pastry, pizza, and pasta), are enjoying an increasing popularity among consumers as convenient, nutritious, low-processed, natural, and healthy food.

The consumption of functional foods with therapeutic and health-promoting effects support the new consumer demands. In general, consumers are already acquainted with the concepts of prebiotics and probiotics due to their intensive promotion by the pharmaceutical, nutraceutical, and agri-food industries. Simultaneously, healthy approaches are needed to overcome the massive antibiotic drugs administration and their side-effects on the colon’s microbiota of mammals [1].

Probiotics are defined as living microorganisms able to exert beneficial effect on consumers’ health. Thus, probiotics should be constantly included in the diet in order to observe health improvements after long-term consumption. Prebiotics, on the other hand, known also as dietary fibers are carbohydrate-based substrates selectively fermented by probiotics in the colon [2,3,4].

New concepts of paraprobiotics and postbiotics are intensively studied, paraprobiotics being defined as killed or “ghost” probiotics and postbiotics being any water-soluble compound biosynthesized by probiotics, including Lactic Acid Bacteria (LAB) strains, during fermentations, or any other type of by-products from the bacterial cells after their lysis by physical, chemical, and/or enzymatic agents that possess health-promoting effects to the host [5,6,7,8,9,10]. The living cells are not a necessary condition to exert the beneficial therapeutic effects on consumers. In addition to the nutritional attributes, the functionalization of foods by postbiotics increases the sensorial and technological properties, as well as their shelf life by reducing the microbial contamination in some manufacturing processes and during storage [6,9,10].

The beneficial effects of prebiotics, probiotics, postbiotics, and paraprobiotics could be maximized in complex functional foods, that include all the above-mentioned bioactive compounds, consumers being interested in this kind of products [11,12].

Furthermore, there is a correlation between the gastro-intestinal microbiota and a healthy lifestyle due to the fact that any perturbation on the typical gastro-intestinal microbiome may lead to unwanted immune responses that, ultimately, may degenerate into health imbalances and diseases [1,13,14]. Despite the existence of some innovative therapies capable to maintain a healthy intestinal microbiota, one of the most common disorders found in humans is celiac disease (CD); the mechanisms behind this condition are still not entirely elucidated. Nevertheless, it is known that CD is influenced by genetic and environmental factors and also by long exposures to gluten-containing foods [15].

People suffering from irritable bowel diseases must include in their diets only gluten-free foods [16,17]. Moreover, they also often suffer from unbalanced diets, such as the lack of dietary fiber and inefficient mineral and other important nutrient absorption (e.g., iron, folic acid, calcium, and fat-soluble vitamins) [18,19]. Examples of gluten-free foods are milk, butter, cheese, fruits and vegetables, meat, fish, poultry, eggs, beans, seeds, nuts, corn, and rice. Gluten-free baking products are not as popular as their gluten-containing counterparts; usually, the former are more expensive and present several disadvantages related to technological, sensorial, and nutritional features. Food scientists try to find innovative solutions to create new gluten-free products such as gluten-free breads, cookies and biscuits, pasta, and beverages [20,21]. To that purpose, healthier gluten-free products could be obtained by innovative approaches that involve the unconventional flours of quinoa, amaranth, and buckwheat or by exploiting the biochemical properties of valuable starter cultures used for substrate fermentations [22,23,24,25,26]. In fact, due to the unbalanced diet based on strict gluten-free foods, enrichment of gluten-free baked goods with proteins, dietary fibers, and other ingredients has emerged. Some examples are the incorporation of inulin (a polysaccharide with prebiotic properties) or dairy powders with high protein and low lactose content (e.g., sodium caseinate and milk protein isolate). Common ingredients of the recipes for gluten-free breads and baking goods are corn, potato, tapioca, and brown or white rice flours or starches. Oat is appropriate for most CD patients, and its interest lies in the high nutritional quality—in particular, its total dietary fiber and β-glucan content. Wheat, rye, barley, triticale, spelt, kamut (and possibly oat) are examples of inappropriate cereals for celiac patients, since the hydrolysis of their prolamins during digestion leads to immune responses and subsequent inflammation of the small intestine. Oat was approved as an ingredient in the gluten-free labelled products in Europe, but its safety upon CD patients is still not entirely resolved. On the contrary, the cereals rice, maize, sorghum, millet, teff, and ragi, as well as the aforementioned pseudocereals, are suitable for celiac patients, thus being susceptible of being incorporated in recipes of gluten-free baking products [25,26].

Gluten is a proteinaceous material, which can be separated from flour when the starch and other minor compounds are removed by washing with water [27,28]. It contains 75–86% protein (on a dry basis), and the remainder is constituted by carbohydrates and lipids. Gluten is responsible for important structural properties exhibited by baking doughs, and its removal or absence results in major technological problems in the bakery industry, since gluten-free doughs are unable to develop a protein network with good baking characteristics. The sourdough biotechnology, including long-term (or slow) fermentations with selected starter cultures, may represent key solutions to overcome the technological handicaps found in gluten-free baking. For example, the production of organic acids by LAB during sourdough fermentation improves gas retention in doughs, can induce antimicrobial effects against spoilage microorganisms, activates endogenous flour proteases, increases water binding to proteins and starch granules, helps in the swelling of pentosans, and activates phytases [25,26]. In addition, the inflammatory reactions of gluten can be minimized by the metabolic activity of LAB strains that hydrolyze the substrate proteins into small fractions without side-effects for the CD patients [24,29,30,31]. Lactic acid bacteria (LAB) occur naturally in many fermented foods, such as sourdoughs and dairy products, and both the microorganisms and their metabolites are classified as Generally Regarded as Safe (GRAS) and Qualified Presumption of Safety (QPS)—thus being considered as non-toxic and food-grade microorganisms [32,33].

Pseudocereals and legumes are valuable sources of proteins, fats, and dietary fibers which can be used for novel gluten-free food products formulation. Their functional properties can be improved by lactic acid fermentation, a process which also enhances the nutritive and sensorial characteristics of the final fermented products. It can be used simply to decrease the risk of cross-contaminations with traces of gluten in food products. As previously mentioned, sourdough LAB is a source of proteolytic enzymes activated by acid production and, subsequently, is likely to be able to eliminate gluten toxicity during breadmaking. In fact, some lactobacilli possess a high potential to degrade Pro- and Gln-rich gliadin oligopeptides in wheat, which are responsible for the human inflammatory responses as a reaction to gluten ingestion [25,26]. Moreover, the lactic acid (sourdough) fermentation enhances the sensorial attributes of the baking products, and its proteolytic activity, in particular, can be efficiently used as well to improve the texture and to delay staling in gluten-free baking breads. Furthermore, the phytic acid and other antinutritional factors from the cereal seeds are reduced by specific enzymes (phytases), thus resulting in a higher bioavailability of important minerals in the fermented products [34,35,36,37,38].

Unlike wheat doughs, where addition of sourdough is optional, the absence of gluten in rye flour makes the addition of sourdough a prerequisite to achieve good baking features. Sourdough fermentation is associated with the development of desired tastes and aroma and extension of shelf-life, as well as numerous nutritional and technological improvements during fermentation and baking. Traditional processes for sourdough fermentation are based on the continuous propagation (or back-slopping) of a fermented dough (usually called mother dough, mother sponge, seed dough, or sour ferment) from batch to batch, which enables a good chemical, functional, and microbiological stability and metabolic activity of the sourdoughs, thus contributing to a constant bread quality. Nevertheless, these processes are typically laborious, time-consuming and frequently poorly reproducible, making single or mixed (or co-cultured) microbial starter cultures an attractive alternative for bakeries [25,26,39,40]. Therefore, the traditional processes of sourdough biotechnology are evolving rapidly into novel and more microbiologically stable sourdoughs based on the design of selected microbial starters and with the potential to be applied to a large spectrum of agri-food matrices and not only restricted to the baking industry [41]. Nevertheless, the number of starter cultures commercially available are few and still present important technological limitations, and the knowledge on their microbial dynamics during fermentation is still incipient.

Nowadays, researchers are often particularly focusing their attention on the discovery and characterization of new LAB strains able to biosynthesize postbiotics such as exopolysaccharides (EPS), bioactive peptides, and short-chain fatty acids, in order to exploit their antimicrobial and functional properties in foods [42,43,44,45]. Aiming at increasing the visibility of the newly discovered postbiotics, as well as facilitating to track the available diversity and compare their features, structures and applications with those already reported in other research efforts, specific large-scale databases of postbiotics produced by LAB strains have been created, chiefly EPS-DB for exopolysaccharides and LABiocin for bacteriocins [46,47]. The exploitation of unconventional pseudocereals, such as quinoa and buckwheat, and legumes, such as chickpea, is decisively a hotspot in the scientific field of sourdough biotechnology and breadmaking, not only due to the demands of the agri-food market but also because these raw materials contain numerous bioactive compounds that can be very useful in as many fields as cancer treatments, combating cellular oxidative stress, or contributing efficiently to the nutritional improvement, as well as agri-food fortification and functionalization [48,49]. Moreover, the selection of LAB strains to further design starter cultures must also consider important features such as the preservation technologies and overall nutritional-functional aspects of the final fermented products [50,51]. Finally, the research studies on this subject should also take into consideration the valorization of the cereal-based by-products, residues, and food wastes and, eventually, its incorporation into new value chains, thus promoting the rational use of natural resources and increasing the environmental sustainability and economic circularity of agri-food systems in agreement with the guidelines expressed in the 2030 Agenda for Sustainable Development [52]. The main aim of this research work was to preliminarily evaluate the technological and functional properties of some wild strains of LAB, isolated from a wide range of natural resources, specifically their acidification capacity, the ability to produce exopolysaccharides, and the antimicrobial capacity against typical food spoilage molds and bacteria. Based on these criteria, as well as on the stability of the antimicrobial activity after thermal and pH treatments of the fermented products, the four best LAB candidates were selected to be further tested as multi-strain starters for gluten-free sourdough production with enhanced functionality.

## 2. Materials and Methods

### 2.1. Materials, Chemicals, and Feedstocks

Raw materials for the prospection and isolation of LAB, depicted in Table 1, were purchased from local Romanian market stores. The samples were transported to the laboratory in plastic bags at room temperature or under refrigerating conditions and stored at −80 °C before use. All the chemicals, reagents, and commercial culture media were purchased from Sigma-Aldrich (Steinheim, Germany).

### 2.2. Prospection of Microorganisms and Culture Conditions

#### 2.2.1. Enrichment and Fermentation Procedures for Microbial Prospection of Lactic Acid Bacteria

The LAB strains were isolated from various raw-materials (Table 1) following multiple protocols of fermentation or microbial enrichment. In some cases, the fermentation/enrichment of the raw samples were undertaken in sterile sample beakers, by mixing 9 mL of de Man, Rogosa, and Sharpe (MRS) broth or Rogosa broth with 1 g or 1 mL of sample and incubating under aerobic batch conditions for 72 h at 37 °C in an incubator (Binder BF4000, Tuttlingen, Germany). The sample beakers containing whey, fermented wheat bran, white beans, soil, Emmental cheese, red lentils, millets, sesame, and hemp seeds were shaken a few times by hand throughout the fermentation time.

In the case of the raw samples of some cereal, legume, and pseudocereal flours (see Table 1), e.g., wheat flours, quinoa, buckwheat, and chickpea, spontaneous type I sourdough fermentations were undertaken. To that purpose, the flour sample was mixed with tap water, at room temperature, in the ratio 1:1 (*w*_sample_/*v*_water_). The ingredients were mixed with a sterile spatula and the resulting homogenous baking dough let to stand and ferment under controlled conditions in an incubator at 37 °C for 24 h. The resulting sourdough was propagated (backslopped) one time by mixing with a mixture of the same flour and water in the proportion of 20 g sourdough to 40 g of flour and 40 mL of tap water.

#### 2.2.2. Microbiological Growth, Isolation, and Purification of Presumptive Lactic Acid Bacteria

Depending on the used protocol, the fermented broth media and decimal dilutions from sourdoughs were used for further inoculation, growth, isolation, and purification of LAB. Decimal dilutions were obtained using a sterile 0.9% (*w*/*v*) NaCl saline solution.

The inoculation of each of the previous samples was carried out with an inoculation loop by the streak-plate technique into Petri dishes of MRS agar supplemented with 1% (*w*/*v*) CaCO_3_ as well as with 150 mg/L of cycloheximide, so as to prevent the growth of yeasts and molds. Inoculated MRS agar Petri dishes were further incubated at 37 °C for 48–72 h under aerobiosis.

After incubation, single colonies surrounded by a clear zone (halo), were selected and streaked in MRS agar and incubated under the same conditions in order to obtain pure cultures. When necessary, the isolates were properly purified by sub-culturing on MRS agar. Microscopic observation of fresh cells and Gram staining of the cells was carried out to verify the purity of the microbial isolates.

#### 2.2.3. Microbiological Identification of Presumptive Lactic Acid Bacteria to the Genus Level

Some morphological and biochemical assays were performed in order to establish the taxonomy of the isolated strains. Thus, the morphological examination was performed with a phase contrast microscope (Olympus, Hamburg, Germany). The Gram staining was done following the standard procedure described by Collins and Lyne (2004) [53] using a smear with a single colony of each presumptive LAB strain on a glass slide.

For the catalase test, the protocol used by Pyar and Peh (2013) [54] was followed. A single colony of presumptive LAB was spread on a glass slide, and a drop of 3% H_2_O_2_ was poured. The effervescence of the oxygen on the slides was considered positive. For the urease test, the culture medium proposed by Foroudandeh et al. (2010) [55] was used. Briefly, the presumptive LAB strains were inoculated on Petri dishes containing 1 g/L peptone, 5 g/L NaCl, 2 g/L mono-potassium phosphate, 1 g/L glucose, 20 g/L urea, 0.1 g/L phenol red, and 15 g/L agar and afterwards incubated at 37 °C for 72 h. The plates that displayed a pink color at the end of the incubation time were considered positive. The rod-shaped isolates that were Gram-positive, catalase- and urease-negative were included in the further tests and preliminarily classified as *Lactobacillus* spp. The Gram-positive, catalase- and urease-negative cocci were preliminarily classified as *Leuconostoc* spp. [56,57,58]. Gram-positive, catalase- and urease-negative ovoid cocci associated in pairs or chains were preliminarily classified as *Streptococcus* spp., and the spherical cocci associated in tetrades were preliminarily classified as *Pediococcus* spp. [59,60].

#### 2.2.4. Maintenance and Reactivation of Pure Cultures of Presumptive Lactic Acid Bacteria

The pure LAB cultures were grown under sterile conditions in MRS broth at 37 °C for 72 h, and stored at −80 °C in an ultra-freezer (Angelantoni, Cimacolle, Italy) in sterile capped plastic tubes (Eppendorf, Vienna, Austria) with 40% (*v*/*v*) glycerol solution until further use [61,62]. All the newly isolated LAB strains were included into the Microorganisms Collection of Dunărea de Jos University of Galați (acronym: MIUG), Galati, Romania. Prior to the characterization studies, the cryopreserved LAB strains were reactivated by cultivation in sample tubes with 9 mL of MRS broth at 37 °C for 48 h under aerobic conditions. Furthermore, 2% (*v*/*v*) fresh inoculum containing approximately 10^9^ CFU/mL (OD_600nm_ = 1.8) was used in the subsequent metabolic assays according to the method reported by Lepczyńska and Dzika (2019) [63], slightly modified. The optical density (OD) of the LAB inoculum was established at a value of 1.8 spectrophotometrically (Libra S22 UV-VIS, Biochrom, Cambridge, UK), using sterile MRS broth as the control.

#### 2.2.5. Microbiological Growth, Isolation, and Purification of Indicator Microorganisms

The indicator microorganisms (molds and bacteria) for the antimicrobial activity assay were isolated from different foods and raw sources, following similar procedures as those described elsewhere [56,64,65,66].

A *Penicillium* spp. strain was isolated from moldy wheat bread as follows: the mold spores were collected from the surface of the bread and streaked on Potato Glucose Agar according to the procedures described previously [65,67]. *Aspergillus niger* and *Aspergillus flavus* strains were isolated from quinoa microbiota by spreading some seeds on Potato Glucose Agar dishes [66] and further incubated (POL-EKO APARATURA, Poland) at 25 °C for 3–5 days. As described previously in Section 2.2.2, repeated sub-culturing by streak plate technique was carried out until pure cultures of molds were obtained. The pure mold spores were collected afterwards from Potato Glucose Agar slants in sterile 0.9% (*w*/*v*) NaCl saline solution by stirring with a vortex mixer (Biosan, Riga, Latvia). The spore’s suspension was then mixed at a proportion of 1:1 (*v*/*v*) with a 40% (*v*/*v*) glycerol solution in sterile capped plastic tubes and stored at −80 °C until further use.

Finally, the endospore-forming Gram-positive rods *Bacillus* spp. strain was isolated from the quinoa seeds microbiota following the procedures described before [64]. Briefly, 1–5 g of seeds were suspended in 0.9% (*w*/*v*) NaCl saline solution, stirred in vortex mixer and subjected to heat treatment of 80 °C for 10 min on a water bath to destroy any vegetative forms and keep only the spores. After cooling, 1 mL of the previous solution was inoculated by spread and streak plate techniques on Petri dishes of Plate Count Agar. After an incubation at 37 °C for 48 h, pure cultures were obtained by successive sub-culturing using the streak plate technique on the same culture medium, and the purity of the bacterial culture was checked with a phase contrast microscope. The pure culture of the indicator bacteria was grown under sterile conditions on Plate Count Agar slants at 37 °C for 48 h in aerobiosis conditions. Gram-positive rods, catalase- and urease-negative, were selected and preliminarily classified as *Bacillus* spp. [68]. For the stock cultures’ preservation, 9 mL of sterile saline solution (0.9% NaCl) was poured into the Plate Count Agar slants, and the bacterial biomass was collected from the surface of the medium with a sterile inoculation loop. The bacterial suspension was then mixed with 40% (*v*/*v*) glycerol solution in a ratio of 1:1 (*v*/*v*) and stored at −80 °C in sterile capped plastic tubes (Eppendorf, Vienna, Austria) until further use.

### 2.3. Evaluation of the Capacity of Presumptive Lactic Acid Bacteria to Produce Exopolysaccharides

The capacity of the newly and pure LAB strains to produce exopolysaccharides was evaluated using the methodologies described by Bachtarzi et al. (2019) [69] and Zhao et al. (2019) [70]. Briefly, the biomass of a single colony of lactic acid bacteria strains previously grown on MRS agar dishes for 48 h at 37 °C in aerobiosis was used for a preliminary sterile cultivation by the streak-plate technique into Petri dishes containing modified MRS agar (MRS supplemented with 50 g/L sucrose and 5 g/L glucose). After the incubation at 37 °C for 72 h, the visual appearance of the colonies was evaluated. The slimy, mucoid colonies of the presumptive LAB strains, as well as those colonies that formed wires to the touch with a sterile loop were selected for further tests.

Moreover, a volume of 0.18 μL of each selected LAB strain previously reactivated in MRS broth was then aseptically inoculated in sterile glass tubes containing 9 mL of modified MRS broth (2% *v*_LAB_/*v*_medium_) and incubated at 37 °C for 72 h under aerobic conditions. Afterwards, the bacterial cells were discharged by centrifugation (Hettich Universal 320R, Tuttlingen, Germany) at 6000 rpm and 4 ˚C for 10 min, and the content of extracellular exopolysaccharides was determined in the collected supernatant by the phenol-sulfuric acid assay using glucose as a standard, as described elsewhere [71,72,73]. Succinctly, 1 mL of cell-free supernatant (CFS) was mixed with 1 mL of 5% (*w*/*v*) phenol solution in distilled water. The mixture was thoroughly vortexed and then 5 mL of 96% (*v*/*v*) H_2_SO_4_ aqueous solution was added. After mixing in the vortex mixer, all the reagents and samples were set aside at room temperature for 30 min. The absorbances of the samples were determined spectrophotometrically at the visible wavelength of 490 nm and taking into consideration the dilution factor of 100. The results were expressed as milligram of total sugars per milliliter of medium.

### 2.4. Evaluation of the Acidification Capacity of Presumptive Lactic Acid Bacteria

The selected pure strains of the new LAB were assessed for their ability to produce organic acids in sterile broths extracted from the flours of the pseudocereals quinoa and buckwheat and the vegetable chickpea, following the procedures described previously [74] with slight modifications. Chickpea, quinoa, and buckwheat were ground in a coffee mill (Bosch, Cluj, Romania) before sampling. Briefly, the pseudocereal or legume flours (5%, *w*/*v*) were mixed with distilled water on a rotary shaker (Lab Companion SI-300, GMI, Minneapolis, MN, USA) at 25 °C, 100 rpm for 30 min. Then the liquid (supernatant) fraction was filtered under vacuum using Whatman no. 1 filter paper (Maidstone, UK) and then sterilized (Panasonic MLS-3871L, Bucharest, Romania) at 121 °C for 15 min. The pure LAB strains were reactivated, under aerobiosis, in MRS broth at 37 °C for 48 h, as previously described.

For the fermentation assays, 150 mL of sterile flour extract broths in a 300 mL Erlenmeyer were inoculated, under aseptic conditions, with 3 mL of the previous fresh pure LAB strains, giving rise to the ratio of 2% (*v*_LAB_/*v*_extract_). Throughout the 72 h of aerobic fermentation at 37 °C, the total titratable acidity (TTA) was determined with an automatic titrator (TitroLine Easy, Schott Instruments, Mainz, Germany), at the beginning of the flour extract fermentation (t_0_) and every 24 h for 3 days. Furthermore, for the TTA determination, a volume of 10 mL of the fermented sample was mixed with 90 mL of distilled water, and the results were expressed as the volume of NaOH 0.1N (mL) required to reach the pH value of 8.5 [75].

### 2.5. Evaluation of the Antimicrobial Properties of Flour Extracts Fermented with Presumptive Lactic Acid Bacteria

The obtained fermented samples (see Section 2.4, were assayed for their antimicrobial properties against indicator bacterial and mold strains obtained as described above. The fermentations were carried out with the all the newly isolated LAB strains (Table 1) on the sample extracts derived from chickpea, quinoa, and buckwheat flours, as described earlier on.

#### 2.5.1. Antifungal Activity by Agar Well Diffusion Assay

The strains of *Aspergillus niger*, *Aspergillus flavus*, and *Penicillium* spp. were used as the indicator molds for the antifungal activity in agar well diffusion assays. The mold strains were grown on Potato Glucose Agar slants at 25 ˚C for 96 h, and the spore forms were further collected in sterile saline (0.9% NaCl) solution supplemented with 0.1% (*v*/*v*) Tween 80, according to the slightly modified methodologies of Cizeikiene et al. (2013) [76] and Garcia-Cela et al. (2020) [77]. In each Petri dish, a volume of 20 mL of melted Potato Glucose Agar (at approximately 50 °C) was poured previously inoculated with the appropriate volume of each suspension of molds to reach a concentration of approzimately 10^5^ spores/mL, counted using a Thoma counting chamber. After the solidification of the media, several wells were created in the solid medium, and 50 µL of the supernatant (without cells) of each fermented flour extract, obtained after centrifugation at 6000 rpm for 10 min at 4 °C (see Section 2.4), was aseptically poured into the agar wells. After incubation for 96 h at 25 °C under aerobiosis, the antifungal activity was evaluated through the following categorical scale: (–) no inhibition; (+) inhibition of spore formation; (++) inhibition of spore formation with a small zone of inhibition around the wells; and (+++) inhibition of the mycelium’s growth around the wells.

#### 2.5.2. Antibacterial Activity

The strain of *Bacillus* spp., used as indicator, was reactivated in Nutrient Broth (1 g/L meat extract, 2 g/L yeast extract, 5 g/L peptone, 5 g/L sodium chloride, pH 7.4) at 37 °C overnight. For the agar well diffusion assays, a volume of 100 µL of the previous overnight inoculum (OD_560 nm_ = 0.08) was used for inoculation into Petri dishes with Plate Count Agar through the surface spread plate technique. Afterwards, wells were created and 50 µL of the supernatant of each fermented flour extract (see Section 2.4 and Section 2.5.1) were aseptically poured into the agar wells. After an incubation at 37˚C for 24 h under aerobiosis, the growth inhibition zone/halo diameters were determined [78].

#### 2.5.3. Stability of the Antimicrobial Capacity of the Fermented Flour Extracts after Undergoing Thermal and Acidification Treatments

The stability of the antimicrobial capacity of the fermented flour extracts with the selected LAB strains, after being subjected to different temperatures and acidification levels, was assessed. To that purpose, the pure LAB strains were cultivated aerobically on sample tubes with MRS broth at 37 ˚C for 48 h, using the methodologies previously described (see Section 2.2.4.). A volume of 150 mL of the sterile flour extracts (Section 2.4) was aerobically fermented in Erlenmeyer flasks at 37 °C for 72 h using 3 mL of inoculum. After 72 h of fermentation, the microbial cells were removed by centrifugation (6000 rpm, 10 min, 4 °C), thus resulting cell-free supernatants (see Section 2.5.1). The stability of the antibacterial and antifungal compounds from the CFS was tested under different pH values (3.5, 5.5, and 7.5) and temperature (60, 80, and 121 °C for 15 min) conditions. All the samples, acidified and thermally treated, were sterilized by filtration trough 0.22 µm filters (Bio Basic, Markham, Canada) right after the adjustment of pH or right after cooling (approximately 40 °C). Then the filter-sterilized samples were used in the antimicrobial assays [79,80,81] through the following methodologies.

##### Antifungal Activity of Fermented Flour Extracts after Undergoing Thermal and Acidification Treatments

The indicator strains of *Aspergillus niger*, *Aspergillus flavus*, and *Penicillium* spp. were used for the antifungal activity of the treated CFS evaluation. The fungal indicator strains were grown on Potato Glucose Agar slants as previously described (see Section 2.5.1). The melted Potato Glucose Agar (at approximately 50 °C) medium was supplemented with 5% (*v*/*v*) CFS, treated as mentioned above (see Section 2.5.3) and then poured into Petri dishes. After the solidification of the medium, 10 μL of the fungal spore suspension containing approximately 10^5^ spores/mL were spotted into the centers of the dishes. The CFS from the sterile flour extracts (unfermented) were used for the control samples. After the incubation of the Petri dishes for 96 h at 25 °C, the inhibition ratio was calculated using Equation (1):

(1) $$I=\frac{A_{C}-A_{T}}{A_{C}}\times \;100$$

where I is the growth inhibition percentage; A_C_ is the diameter (in mm) of the mycelial growth in the control sample, and A_T_ is the diameter (in mm) of the mycelial growth in the medium supplemented with the treated sample [79,82].

##### Antibacterial Activity of Fermented Flour Extracts after Undergoing Thermal and Acidification Treatments

The CFS subjected to different temperatures and acidification levels (Section 2.5.3) were used to evaluate the antibacterial activity against the bacterial food spoilage indicator microorganism *Bacillus* spp. following the methodologies previously described (Section 2.5.2).

### 2.6. Statistical Analysis

The experiments were carried out in triplicate, and the results were expressed as mean value ± standard deviation (SD) for three independent measures (*n* = 3). Furthermore, regarding the statistical treatment, a Principal Component Analysis (PCA) of the experimental data was performed using Minitab 17 (version 1.0, Minitab LLC, Pennsylvania, United States) with the following variables: (1) Antifungal activity of the chickpea flour extract against *Aspergillus niger*, (2) Antifungal activity of the chickpea flour extract against *Aspergillus flavus*, (3) Antifungal activity of the chickpea flour extract against *Penicillium* spp., (4) Antibacterial activity of the chickpea flour extract against *Bacillus* spp., (5) Acidification capacity measured as TTA after a 72 h fermentation on the chickpea flour extract, (6) Antifungal activity of the quinoa flour extract against *Aspergillus niger*, (7) Antifungal activity of the quinoa flour extract against *Aspergillus flavus*, (8) Antifungal activity of the quinoa flour extract against *Penicillium* spp., (9) Antibacterial activity of the quinoa flour extract against *Bacillus* spp., (10) Acidification capacity measured as TTA after a 72 h fermentation on the quinoa flour extract, (11) Antifungal activity of the buckwheat flour extract against *Aspergillus niger*, (12) Antifungal activity of the buckwheat flour extract against *Penicillium* spp., (13) Antibacterial activity of the buckwheat flour extract against *Bacillus* spp., (14) Acidification capacity measured as TTA after a 72 h fermentation on the buckwheat flour extract, (15) Production of exopolysaccharides.

## 3. Results

### 3.1. Isolation of Wild Presumptive Lactic Acid Bacteria

LAB strains were isolated from several food materials, viz., cereals, pseudocereals, vegetable seeds, sourdoughs, and fermented products (Table 1). In addition, soil samples were also a valuable source of LAB, resulting in the isolation of three different strains. Furthermore, the LAB strains isolated from wheat flour, buckwheat, chickpea, and quinoa were also included in the following studies.

The nineteen LAB strains depicted in Table 1 were subjected to a morphological and biochemical characterization, allowing their identification and grouping at the genus level [56,57,58,59,60]. The isolates were grouped into 4 distinct genera as follows: twelve isolates belonging to the regular non-spore forming rods *Lactobacillus* spp., four isolates belonging to the Gram-positive ovoid cocci *Leuconostoc* spp., two isolates belonging to *Pediococcus* spp., and one strain belonging to *Streptococcus* spp.

### 3.2. Characterization of the Technological and Functional Properties of the Presumptive LAB Strains

#### 3.2.1. Production of Exopolysaccharides by Presumptive Lactic Acid Bacteria

Amidst other reasons, in the food industry, LAB strains are potentially interesting because of the capacity of some of them to biosynthesize exopolysaccharides with multiple nutritional and technological implications [83]. Among all the nineteen tested LAB strains, Table 2 lists only the nine strains that produced exopolysaccharides in amounts over 10 mg/mL when grown in an MRS broth, supplemented with 5 g/L glucose and 50 g/L sucrose. The exopolysaccharides quantified through the phenol-sulfuric acid assay after 72 h of aerobic fermentation at 37 °C ranged between 11.307–23.193 mg/mL for these most productive strains.

#### 3.2.2. Acidification Capacity of the Presumptive Lactic Acid Bacteria

**Figure 1:**
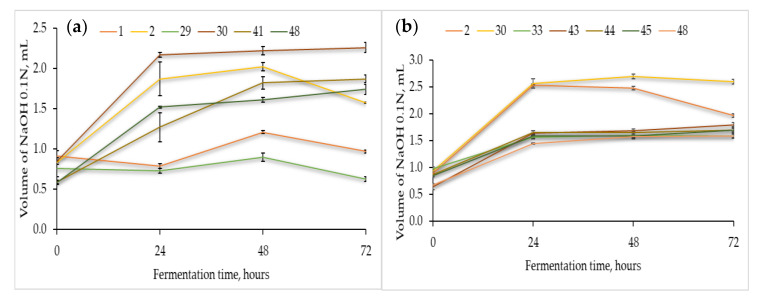

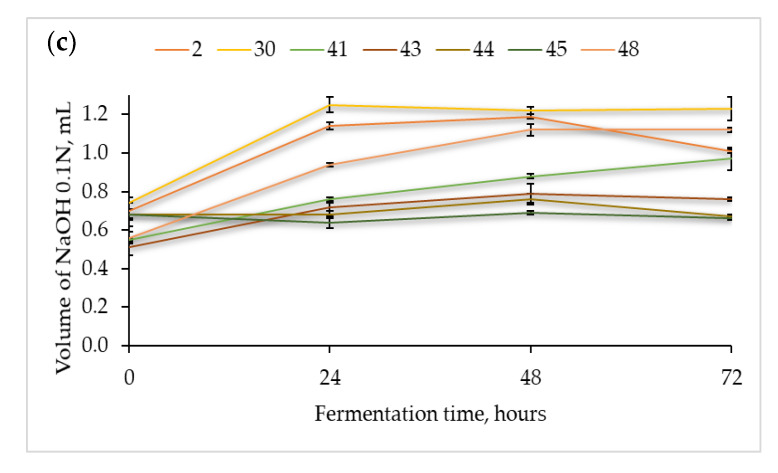
Acidification capacity (average ± standard deviation), measured in triplicate as total titratable acidity (TTA) of the selected presumptive Lactic Acid Bacteria when cultivated in sterile flour extracts of (**a**) chickpea, (**b**) quinoa, and (**c**) buckwheat at 37 °C for 72 h.

The acidification capacity of all the LAB strains (Table 1) was determined by their cultivation in sterile flour extracts of chickpea, quinoa, and buckwheat (Figure 1). As expected, the acidification capacity differed with the fermentation substrate and LAB strain. Figure 1 also shows that most of the selected strains needed 24 h of adaptation until significant rates of acid production were reached.

In the chickpea flour extract (Figure 1a), strain no. 30 reached the maximum acidification capacity after 48 h of fermentation at 37 °C. The remaining strains, viz., no. 2, 41, and 48, reached the maximum acidification potential after 48–72 h of fermentation. Thus, strains 30 and 2 were responsible for the fastest acidification of the chickpea flour extract.

Likewise, the fermentations in the quinoa (Figure 1b) and buckwheat flour extracts (Figure 1c) showed that strain no. 30 was the most productive, and the maximum values were essentially reached after 48 and 24 h, respectively. Different levels of TTA were found in the three matrices, for instance, for strain no. 30, the maximum volume of NaOH was approximately 2.2, 2.7, and 1.2 mL in the fermentations with chickpea (72h), quinoa (48h), and buckwheat (24h), respectively, hence emphasizing the effect of the food matrixes in the acid production capacity by the same LAB strain. Finally, in the buckwheat flour extract (Figure 1c), strains no. 2, 48, and 41 showed good acidification capacity.

As unfolded in Figure 1, some of the LAB strains were able to adapt faster to the lactic acid fermentation of the pseudocereal and legume extracts broths. In particular, strains no. 30 and no. 2 exhibited a good acidification potential after 48 and 72 h of fermentation, respectively. These results emphasize the diversity of metabolic traits among different LAB strains as well as the dependence on the available nutrients of different matrixes.

#### 3.2.3. Antimicrobial Properties of Flours Fermented with Presumptive Lactic Acid Bacteria

According to Table 3, the antifungal activity differs among the LAB strains and substantial differences could also be observed among the type of fermentation matrix (i.e., sterile flour extracts of chickpea, quinoa, and buckwheat). The chickpea flour extract fermented with the LAB strain no. 1 showed a good antifungal activity against spores forming *Aspergillus niger*. Moreover, the chickpea flour extract fermented with the LAB strains no. 13 and no. 20 displayed a weak antifungal activity against *Aspergillus niger*. For the same fermentation substrate, a very good inhibition was observed for LAB strain no. 48 against the *Aspergillus flavus* sporulation. Nevertheless, the chickpea flour extract fermented with LAB strains no. 20, 24, 29, 41, 43, and 44 demonstrated a weak inhibition activity against *Penicillium* spp.

In the fermentation of quinoa flour extract with the LAB strains no. 20, 24, 29, 30, 31, and 33 a high inhibition capacity against *Aspergillus niger* was observed. On the contrary, the fermented quinoa flour extract with the LAB strain no. 48 revealed the absence of inhibition capacity against *Aspergillus niger*, although a weak inhibitory effect was observed against *Penicillium* spp.

Moreover, none of the fermented buckwheat flour extracts exhibited inhibition capacity against *Aspergillus flavus*.

The LAB strain no. 24 revealed a very good antibacterial activity against *Bacillus* spp. (inhibition zone diameter > 21 mm) in any of the tested fermentation substrates (chickpea, quinoa, and buckwheat). Moreover, all the fermented extracts with the strains no. 1, 2, 30, 33, 36, and 43 displayed a very good antibacterial activity against the endospore forming Gram-positive rods *Bacillus* spp. The buckwheat and quinoa flour extracts fermented with the strains no. 29, 44, and 48 possessed the best antibacterial activity.

#### 3.2.4. Principal Component Analysis to Discriminate the Most Relevant Factors and Presumptive Lactic Acid Bacteria strains for Further Application in Non-Gluten Sourdough Fermentations

**Figure 2:**
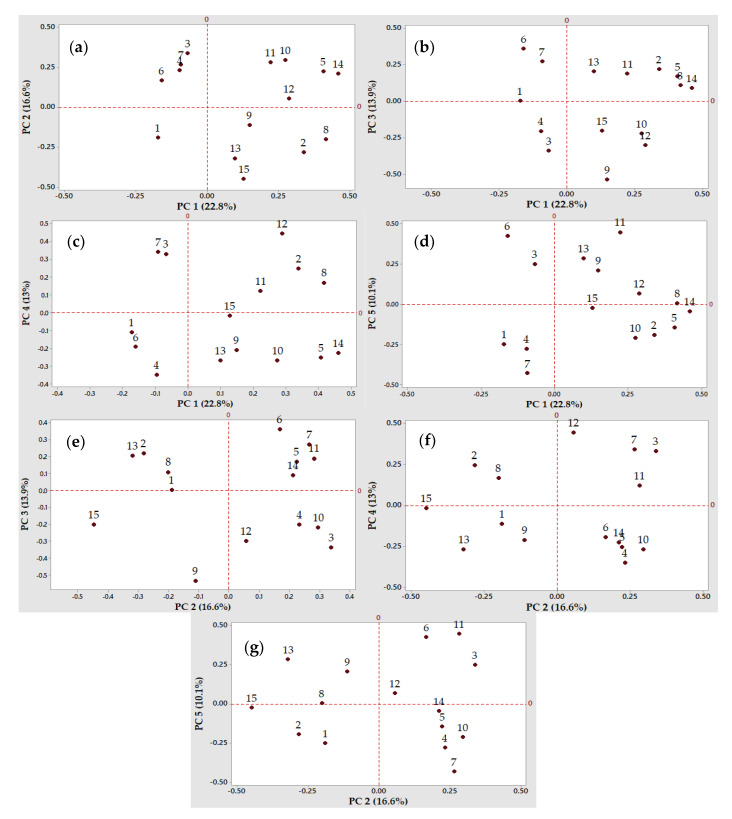
Two-dimensional loading plots of the Principal Component Analysis (PCA). Projection of the variables into the principal component (PC) 1 to 5 (cumulative percent variability in parentheses): (**a**) PC 1 and PC 2 (39.4%); (**b**) PC 1 and PC 3 (36.7%); (**c**) PC 1 and PC 4 (35.8%); (**d**) PC 1 and PC 5 (32.9%); (**e**) PC 2 and PC 3 (30.5%); (**f**) PC 2 and PC 4 (29.4%); and (**g**) PC 2 and PC 5 (26.7%). The numbers in the plots refer to the identification of variables.

**Figure 3:**
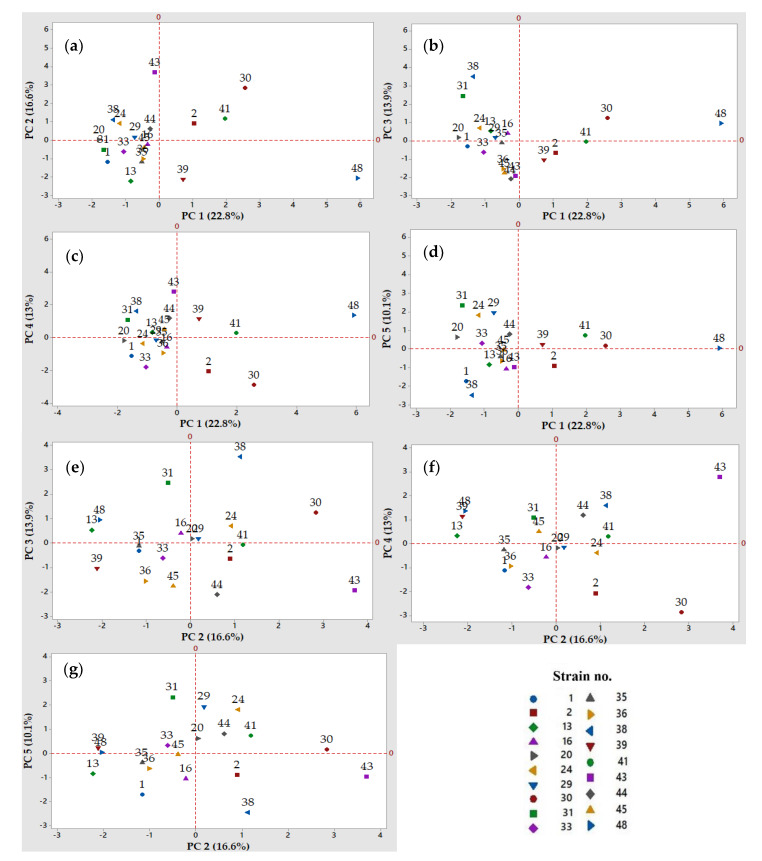
Two-dimensional score plots of the Principal Component Analysis (PCA). Projection of the observations (LAB strains) into the principal component (PC) 1 to 5 (cumulative percent variability in parentheses), for the independent variables depicted in Figure 2: (**a**) PC 1 and PC 2 (39.4%); (**b**) PC 1 and PC 3 (36.7%); (**c**) PC 1 and PC 4 (35.8%); (**d**) PC 1 and PC 5 (32.9%); (**e**) PC 2 and PC 3 (30.5%); (**f**) PC 2 and PC 4 (29.4%); and (**g**) PC 2 and PC 5 (26.7%). The numbers in the plots refer to the identification of LAB strains.

The main aim of the current research work was to unfold the most favorable LAB strains to be further employed as single or co-cultured (multi-strain) starters in the gluten-free sourdough production. To that purpose, a set of fermentation variables (15) were included in the PCA, as the LAB acidification capacity in the fermentations carried out on chickpea, quinoa, and buckwheat flour extracts, the LAB capacity to produce exopolysaccharides and the antimicrobial (antibacterial and antifungal) activity of the resulting fermented flour extracts. This study highlighted the distribution of the analyzed variables and lactic acid bacteria strains depicted in Figure 2 and Figure 3. The Principal Component Analysis was performed using a correlation matrix without rotation in order to reduce the amount of data and better discriminate the observations (the LAB strains), taking into consideration the same evaluation scale of the results. The first 5 principal components (PC1 to PC5) explained 76.4% of the variation of the data, being selected based on the Kaisher criterion (eigenvalue >1). The quadrants in the two-dimensional plots divided the LAB strains and variables into clusters. In each plot, the variables and the corresponding LAB strains with a positive correlation on both PCs can be observed in quadrant I and quadrant IV. Consequently, in the interaction of PC 1 with the rest of the PCs, it can be observed that LAB strains no. 2, 30, and 48 were part of the clusters with positive impact (Figure 3a–c). This behavior of the abovementioned strains can be justified by the loading plots of the variables depicted in Figure 2a–c. Moreover, along with LAB strains no. 2 and 30, strains no. 20 and 24 were positively correlated with PC 2 in their interactions with PC 3 and PC 5 (Figure 3e,g). On the opposite, the variables negatively correlated with PC 2, PC 3 and PC 4 can be observed in the second respectively third quadrant (Figure 2e,f). Furthermore, the distribution and grouping of the LAB strains into the plots’ quadrants highlighted their different behavior (Figure 3a,f).

Based on the PCA results (Figure 2 and Figure 3), four LAB strains were considered to have a high potential for the gluten-free sourdough fermentations, mainly strains no. 2, 24, 30, and 48. As such, strains no. 2 and 30 belonging to the *Lactobacillus* spp. produced the highest amounts of organic acids that influenced the total titrable acidity (TTA) when chickpea, quinoa, and buckwheat flour extracts were fermented by these presumptive lactic acid bacteria strains (Figure 2a–c,g and Figure 3a–c,g).

Strain no. 24 was also selected as a valuable candidate to produce antimicrobials with antifungal and antibacterial activities, with a variable spectrum of inhibition depending on the fermentation substrate (quinoa, buckwheat or chickpea flour extracts). Strain no. 30 could be considered a good candidate due to its antimicrobials (Figure 2d or Figure 3d) when the quinoa and buckwheat flour extracts were used as fermentation substrates.

Strain 48 belonging to the *Leuconostoc* spp. was selected for its multiple capacities to acidify the chickpea and buckwheat flour extracts, for the exopolysaccharides production, and finally for the antimicrobial properties, all these features being observed on PC 1 (Figure 2a,b) and PC 4 (Figure 2c and Figure 3c).

#### 3.2.5. Stability of the Antimicrobial Properties of Fermented Flour Extracts after Undergoing Thermal and Acidification Treatments

The stability of the antimicrobial compounds after thermal and acidification treatments of the cell-free extracts was assessed by fermentation with two selected LAB strains, specifically with the *Lactobacillus* spp. strain no. 24 and *Leuconostoc* spp. strain no. 48. These two strains were taken into account based also on their biotechnological properties previously highlighted. The stability test results showed that the antifungal activity after the treatments (Table 4) was different, depending on the used fermentation substrates. A correlation between the treatments applied on the fermented samples and their antifungal properties was also observed. Specifically, the chickpea flour extract fermented by strain no. 24 without any additional treatments had an antifungal activity only against the *Penicillium* spp. (I = 5.05%), whereas a higher inhibition ratio (I = 12.12%) against the same mold was calculated for the chickpea flour extract fermented by strain no. 48. Strain no. 24 also showed an antagonistic effect against *Aspergillus niger*, the highest inhibition ratio (I = 20.19%) being calculated for the fermented chickpea flour extract that had a pH = 5.5.

LAB strain no. 24 demonstrated a weak inhibitory activity (I = 0.82%) against *Aspergillus niger* in the absence of additional treatments in the fermented quinoa flour extract. Strain no. 48 demonstrated an inhibition ratio of 6.93% against *Penicillium* spp. when the same extract was used. Furthermore, both strains showed a significant inhibitory activity against *Penicillium* spp. when the fermented quinoa extract was treated at 60 °C for 15 min, the highest inhibition ratio (I = 17.33%) belonging to strain no. 24.

Moreover, antifungal activity was detected for strain no. 24 that was used for the fermentation of the quinoa flour extract that was subjected afterwards to sterilization (121 °C, 15 min). In this case, *Aspergillus niger* was inhibited by the fermented extract obtained with LAB strain no. 24 with a ratio of 7.20%, here concluding that the antifungal activity could be related to other biosynthesized compounds that are stable under those conditions, except the organic acids.

After the fermentation of the buckwheat flour extract for 72 h at 37 °C by both of the selected LAB strains that were tested, i.e., strains no. 24 and 48, respectively, it can be concluded that within the fermented broth, several antifungal compounds exist, molecules that were stable at pH 7.5 with inhibition ratios of 7.02% and 10.53% against *Aspergillus niger*. Furthermore, in the fermented buckwheat broth heated at 80 °C for 15 min, various heat-stable compounds are also present with an inhibitory effect against the *Penicillium* spp., with inhibition ratios of 9.88% and 29.44%, respectively.

The antibacterial activity of the samples fermented by LAB strain no. 24, without any other treatment, was observed only for the fermented quinoa flour extract (Table 5), whereas strain no. 48 could not overcome the growth of *Bacillus* spp. on the fermented buckwheat flour extract. The diameters of the inhibition zones were greater than 20 mm for all the flour extracts fermented by strains no. 24 and no. 48 at 60 °C for 15 min. Therefore, the antibacterial compounds that remained active after the thermal treatment at 121 °C or at the pH values of 3.5, 5.5, and 7.5 were not produced by the lactic acid bacteria strain no. 48 on the fermented buckwheat flour extract.

Taking into account the overall antimicrobial activity, it can be observed that the highest antibacterial activity of the untreated samples (16.67 mm zone of inhibition) was correlated to the highest inhibition ratio (I = 12.12%) against the *Penicillium* spp. of the untreated chickpea flour extract fermented with the lactic acid bacteria strain no. 48. This strain inhibited neither the *Bacillus* spp., nor the fungal strains included in the study of the untreated (only fermented) buckwheat flour extract. Even though the highest percentage of inhibition was calculated for the antifungal activity against *Penicillium* spp. and *Aspergillus niger* by the buckwheat flour extract fermented by strain no. 48 and then subjected to different treatments (80 °C or pH 3.5), the antibacterial activity displayed no positive results. Hence, the *Penicillium* spp. development was inhibited at a percentage of 25.60% when the temperature of 80 °C was applied for 15 min on the quinoa flour extract fermented by LAB strain no. 24. In addition, the sample mentioned above determined a very good inhibition of the *Bacillus* spp. growth under the same conditions.

## 4. Discussion

### 4.1. Selection of Presumptive Lactic Acid Bacteria Strains Based on Their Fermentation Traits, Using Multivariate Analysis

The nineteen wild presumptive LAB strains were isolated from various sources such as fermented products, seeds, cereals, flours, and soil (Table 1). By exploiting the natural microbiota in a wide range of different natural sources, researchers seek to find the greatest possible diversity and to select the most competitive strains taking into consideration different nutritional, technological, and functional features. Taroub et al. (2019) [84] isolated from grapefruit lactic acid bacteria strains belonging to *Pediococcus* spp. and *Lactobacillus* spp. These microorganisms proved to be able to inhibit the *Aspergillus* spp. growth and, therefore, to avoid the production of their mycotoxins. Other lactic acid bacteria strains with antimicrobial effects against several pathogens were found, for instance, in some spontaneous wheat, rye, and sorghum sourdoughs [74,85,86,87,88] or home-made cheeses [89,90]. Furthermore, a study regarding a Chinese sourdough revealed that most of the isolated strains belonged to the *Lactobacillus* spp., followed by *Pediococcus* spp. and *Leuconostoc* spp.—their prevalence was thought to be influenced by the origin of the sourdough [87,91]. Other authors studied different lactic acid bacteria strains originated from Japanese bakeries and their ability to enhance the aroma profile and the overall sensorial properties of the sourdough-baked goods [92]. In this study and others [34,61,93], lactic acid bacteria strains were isolated from millets, chickpea, and quinoa to be further selected according to their most interesting technological features.

Total titratable acidity is a key parameter in the sourdough fermentation because it reveals the extension of the organic acids and other valuable antimicrobial compounds production, which, in turn, plays important roles in the sourdough and bread industry, such as antimicrobial and antioxidant properties which contribute to the final taste and aroma, increase the shelf-life, etc. The values of TTA observed in this work (Figure 1) were not as high as the values reported in other works where the levels exceeded 10 mL of NaOH 0.1 N. Such differences depend on the experimental conditions and in spite of these discrepancies, the values of the pH below 4.0 (data not shown) measured in the samples of fermented chickpea, quinoa, and buckwheat extracts after 72 h at 37 °C are in agreement to those found in the literature [82,91,94,95]. The main technological characteristics of a stable mature sourdough are the pH, total titratable acidity, and the fermentation quotient [39,40], and for future work, the fermentation conditions of the gluten-free sourdoughs with the selected LAB will be optimized in order to increase the fermentation potential. Typically, a well-developed sourdough reaches pH values around 3.5–4.0, and log viable counts of 9 and 6–8 of bacteria and yeasts per gram of dough, respectively [39,40,96,97]

The PCA with all the 15 variables (Figure 2) was used to discriminate and select the most favorable LAB strains (Figure 3) for further gluten-free sourdough production. PCA is a versatile tool to reveal correlations and clusters among variables and observation factors, thus allowing the reduction of complex experimental designs and, ultimately, helping in the discrimination analysis [98]. Multivariate analysis was used in order to select the best-performing lactic acid bacteria strains to formulate a starter culture for sausage fermentation [99] or to determine the existing correlations between specific compounds or ingredients and the sensorial properties of the foods [100,101,102,103,104]. PCA was successfully used to emphasize the microbial diversity of some sourdoughs from different geographical areas [91,105,106,107,108,109,110]. In this study, the analysis of the first five PCs with eigenvalues higher than the unit explained 76.4% of the variance. Based on these PCs, the valuable strains to be included in the next studies were the *Lactobacillus* spp. no. 2, 24, and 30 and the *Leuconostoc* spp. no. 48 due to their superior properties regarding acidification, antimicrobial properties, and exopolysaccharides production.

### 4.2. Capacity of Presumptive Lactic Acid Bacteria to Produce Exopolysaccharides

The screening among all the isolates of the presumptive lactic acid bacteria strains presented in Table 1 allowed the selection of those which have proven to possess a good capacity to produce exopolysaccharides (Table 2). Prior to the determination of the total carbohydrates content by the phenol-sulfuric acid assay, all the isolated strains were screened for the EPS production by cultivation on a modified MRS agar medium in order to evaluate their ropy or mucoid visual appearance. Although this preliminary evaluation introduced a lot of subjectivity, it was not used to exclude any of the lactic acid bacteria strains. As a matter of fact, some studies suggested the absence of a correlation between the ropy or non-ropy appearance and the capacity to metabolize exopolysaccharides, since the ropy or non-ropy appearance is related to the EPS type [111]. For instance, Nachtigall et al. (2020) [112] demonstrated the higher capacity of a non-ropy *Streptococcus termophillus* lactic acid bacteria strain to produce EPS (404 mg glucose equivalents per liter) compared to the ropy *Lactococcus lactis* lactic acid bacteria strain, in particular, 404 against 354 mg of glucose equivalents per liter.

In this research work, the results of the phenol-sulfuric acid assay were used to detect and select the highly EPS-productive strains, even though the ropy visual (and to the touch) appearance is the most commonly used method to select the strains, being usually followed by the precipitation of the EPS and the determination of its sugar content by the phenol-sulfuric acid assay. In a study conducted by Amao et al. (2019) [72], a *Lactobacillus plantarum* strain originating from cassava peel heaps produced more than 10 g/L exopolysaccharides. In the present study, the largest LAB producers, viz., strains no. 36 (*Lactobacillus* spp.) and no. 48 *(Leuconostoc* spp.), yielded more than double of those amounts.

Furthermore, fermented meat, fruits, and dairy products were demonstrated to be good sources for the isolation of *Leuconostoc* strains with interesting capacities to produce exopolysaccharides. In the studies carried out with human breast milk [70,113,114,115,116], *Enterococcus faecalis* and *Lactobacillus* spp. strains yielded amounts of EPS ranging from 380 to 737.3 mg/L. In the studies by Adebayo-Tayo et al. (2018) [117] and Adebayo-Tayo et al. (2020) [118], the EPS production yields were compared between mutant and wild LAB strains, where the wild strain of *Lactobacillus delbrueckii* has been shown to be more effective (5.57 g/L). Nonetheless, in the same study [117] the EPS yields of 5.49 and 5.58 g/L with a wild and mutant strain of *Weissella confusa* were obtained.

The EPS production yields by the presumptive LAB in the current work were frequently higher than those described in the literature, the maximum value (approximately 22 g/L) being attained by strains no. 36 and 48. For future studies, the EPS will be characterized in terms of the type (e.g., glucans, fructans, fructooligosaccharides, etc.) as well as in terms of their structural and functional properties and the fermentation conditions optimized to yield high rates of EPS. Li et al. (2020) [119] evaluated the impact of the inoculum, fermentation time, and temperature on the EPS production by *Lactobacillus paracasei*, having obtained a maximum yield of 932 mg/L. Moreover, the carbon-to-nitrogen ratio among many other growth parameters proved to have a major impact on the amounts and quality of the EPS [120].

### 4.3. Antimicrobial Properties of Fermented Flour Extracts with Presumptive Lactic Acid Bacteria against Typical Spoilage Microorganisms in Bakery Products

Luz et al. (2019) [121] studied the effect of some metabolites produced by two lactic acid bacteria strains, specifically *Lactobacillus plantarum* and *Lactobacillus delbrueckii* ssp. *bulgaricus*, cultivated on wheat dough soluble extracts, and found out that the *Lactobacillus plantarum* strain had a better inhibitory effect against *Penicillium* spp., whereas a weaker inhibition (+) was observed against *Aspergillus niger*. The LAB strains included in this study [121] were further used for the manufacturing of sourdough bread, which is likely to exhibit good resistance against fungal contaminations. Some fermented flour extracts with the selected lactic acid bacteria strains included in the current work, in particular, strains no. 20, 24, 29, 30, and 31, exhibited good inhibitory effect against *Aspergillus niger*, *Penicillium* spp., and *Aspergillus flavus*, used as the indicator fungal strains.

In a study by Bartkiene et al. (2019) [85], lactic acid bacteria strains isolated from a spontaneous rye sourdough and classified as *Leuconostoc mesenteroides*, *Lactobacillus curvatus*, and *Lactobacillus brevis* showed the absence of an inhibitory effect against *Aspergillus fischeri* and a weak inhibition against *Penicillium* spp. On the other hand, in the study by Sun et al. (2020) [122], three LAB strains belonging to *Lactobacillus* spp. displayed good inhibitory capacity against *Aspergillus niger*, although most of the tested strains showed a better inhibition against *Penicillium citrinum*, *Aspergillus flavus*, *Aspergillus fumigatus*, and *Fusarium graminearum*. According to the study reported by Omedi et al. (2019) [82], the microbiota of some exotic fruits have proven to be a good source for the isolation of lactic acid bacteria strains with good antifungal activities against *Aspergillus niger* and *Penicillium chrysogenum*. Nonetheless, the same work [82] demonstrated that the addition of 4% cell-free supernatants previously fermented by *Lactobacillus pentosus* strains could not inhibit *Aspergillus niger*, in contrast to some strains of *Pediococcus pentosaceus*, where reasonable inhibition effects were observed.

In a study led by Dentice Maidana et al. (2020) [123], only two lactic acid bacteria strains identified as *Weissella cibaria* could inhibit the growth of *Bacillus subtilis*.

In the present work, the tested lactic acid bacteria strains showed a very satisfactory inhibition capacity against *Bacillus* spp. The results are in accordance with the studies performed by Kaya and Simsek (2019) [124] and Salomskiene et al. (2019) [125] who reported the inhibitory capacity of several species of *Lactobacillus* against *Bacillus cereus.* In contrast, in the study conducted by Verón et al. (2017) [78], most of the *Lactobacillus* strains could not inhibit the growth of the spoilage bacterial strain *Bacillus* spp.

Another important objective in the present work was to evaluate the antimicrobial properties of the chickpea, quinoa, and buckwheat fermented flour extracts without cells (i.e., cell-free supernatants, CFS) after being submitted to different levels of thermal (temperature 60, 80, and 121 ˚C for 15 min) and acidic (pH of 3.5, 5.5, and 7.5) treatments. The aerobic fermentations of the sterile flour extracts were undertaken with the *Lactobacillus* spp. (strain no. 24) and *Leuconostoc* spp. (strain no. 48) strains for 72 h at 37˚C and the CFS subjected to the abovementioned treatments to ascertain the stability of the antimicrobial compounds on such complex food matrixes.

The results demonstrate that the buckwheat flour extract fermented with lactic acid bacteria strain no. 48 showed a great antifungal activity against *Aspergillus niger*, not only at pH values of 3.5 (I = 13.16%) but also at the pH values of 7.5 (I = 10.53%). A similar behavior of another lactic acid bacteria strain was reported by Varsha et al. (2014) [126], who demonstrated that the antifungal activity of the lactic acid bacteria cell-free supernatants decreased with the increasing pH. In the same work carried out by Varsha et al. (2014), it was shown that in the fermentation medium after sterilization (15 min at 121 °C), antifungal compounds were produced. Similar findings were obtained in the current work with the selected lactic acid bacteria strains. Thus, after the sterilization of the quinoa flour extract fermented with strain no. 24, an important inhibition effect against *Aspergillus niger* (I = 7.20%) was observed. Nevertheless, such antimicrobial compounds represent important technological features to the baking industry. Conversely, the fermented chickpea flour extract by lactic acid bacteria strain no. 48 unfolded an inhibitory ratio of 12.1% and 11.1% against *Penicillium* spp. in the absence of the treatments or when heated at 80 ˚C for 15 min, respectively. In addition, the quinoa flour extract fermented by lactic acid bacteria strain no. 24 that was subjected to a thermal treatment at 80 °C for 15 min exposed an inhibitory ratio above 25% against *Penicillium* spp.

As already mentioned, these preliminary assays on the stability of antimicrobial compounds may suggest the presence of lactic acid bacteria metabolites with antimicrobial effects, such as organic acids, bacteriocins, bacteriocin-like inhibitory substances (BLIS), and others. The biosynthesized metabolites are correlated, on the one hand, to the biochemical characteristics of the lactic acid bacteria and to the composition of the extracts from the studied pseudocereal and legume flours, on the other.

Similar studies can be found in the literature emphasizing the presence of antifungal compounds produced and released in the culture medium by lactic acid bacteria strains after 48 h of fermentation and stable at pH values ranging from 5.5 to 7.5 [127]. In another study, the production of bioactive peptides by lactobacilli with antifungal activity was reported [128]. In the work undertaken by Cizeikiene et al. (2013) [76], the antibacterial activities of some lactic acid bacteria strains characterized by inhibition zones ranging between 14–22 mm were determined in some CFS, whereas smaller inhibition zones were observed when the purified bacteriocins of those lactic acid bacteria strains were tested. In our work, the fermented flour extracts with lactic acid bacteria strains no. 24 and no. 48 maintained their antibacterial activity against *Bacillus* spp. after the thermal and acid treatments.

The antimicrobial activity of the lactic acid bacteria is strain dependent although it also depends on the targeted spoilage microorganism and on many other factors such as the fermentation conditions, type of raw materials, etc., thus explaining the diversity of the results found in the literature. Some of the strains isolated in this work are able to biosynthesize compounds which showed a weak inhibition of *Aspergillus niger* when the CFS with pH = 7.5 was used. In contrast, other researchers reported the absence of inhibitory effects of some species of *Lactobacillus* and *Pediococcus* against *Aspergillus flavus* and *A. carbonarius* when a CFS with pH 7.0 was used [79]. Nonetheless, good inhibition effects against *Aspergillus flavus* and *Aspergillus carbonarius* were found by Missaoui et al. (2019) [129] when fermented products with lactic acid bacteria strains isolated from a traditional fermented product named Zgougou were tested.

Overall, this research effort presents the selection of lactic acid bacteria strains, in particular, strains no. 2, 24, 30, and 48, which demonstrated valuable biotechnological characteristics in terms of acidification properties, exopolysaccharide biosynthesis, and antimicrobial properties that could be employed in the gluten-free sourdough fermentations. These four strains could be used as a multi-strain starter due to the complexity of their biochemical properties. They have the advantage to be adapted to the gluten-free environment and are rich in protein flours originated from pseudocereals and legumes as fermentation substrates. In future research studies, these selected lactic acid bacteria are likely to be used for the optimization of the biotechnological processes for the production of gluten-free sourdough rich in proteins, based on chickpea, quinoa, and buckwheat flours that have valuable technological and functional properties.

## 5. Conclusions

The aim of this research work was the isolation and selection of presumptive lactic acid bacteria wild strains, demonstrating important functional properties that can be subsequently used in gluten-free and plant-based protein-rich sourdough fermentations. Such a selection was based on some key technological and functional features for the sourdough fermentation and breadmaking, particularly the capacity of acidification, the capacity to produce exopolysaccharides, and the antibacterial and antifungal activities.

Among nineteen presumptive LAB strains isolated from several raw materials, four strains demonstrated beneficial characteristics for gluten-free and plant-based protein-rich sourdough production and were therefore selected for future studies. In fact, strains no. 24 and no. 48, presumptively classified as *Lactobacillus* spp. and *Leuconostoc* spp., have demonstrated better performances in terms of the elected technological and functional features. These strains showed good antimicrobial properties. It should also be mentioned that the lactic acid bacteria strain no. 48 was able to produce high amounts of exopolysaccharides and antimicrobial substances. Nevertheless, strains no. 2 and strain no. 30, presumptively classified as *Lactobacillus* spp., exhibited the best acidification potential, although they failed, compared to the other two, at the good antimicrobial capacity.

The reported data is part of a more comprehensive research work that will involve the genotype identification of the isolated microorganisms as well as complementary studies for the phenotype characterization. Furthermore, the future work will involve the optimization of the functional microbial metabolites as well as the optimization of the new sourdough fermentations and formulation for gluten-free and protein-rich plant-based recipes.

## Figures and Tables

**Table 1:** Raw materials used for the isolation of microorganisms and encoding of the presumptive by Lactic Acid Bacteria (LAB) strains for further studies.

Strain No.	MIUG Code *	Source of Isolation	Presumptive Genus
1	MIUG BL 68	Buckwheat seeds	*Lactobacillus* spp.
2	MIUG BL 21	Soil	
13	MIUG BL 57	Millet seeds	
16	MIUG BL 53	White beans	
20	MIUG BL 71	Quinoa seeds	
24	MIUG BL 38	Chickpea seeds	
29	MIUG BL 34	Fermented wheat bran	
30	MIUG BL 24	Whey	
31	MIUG BL 60	Hemp seeds	
33	MIUG BL 25	Wheat flour	*Pediococcus* spp.
35	MIUG BL 27	Soil	*Leuconostoc* spp.
36	MIUG BL 39	Whole wheat flour	*Lactobacillus* spp.
38	MIUG BL 59	Red lentil seeds	
39	MIUG BL 7	Soil	*Pediococcus* spp.
41	MIUG BL 66	Chickpea seeds	*Leuconostoc* spp.
43	MIUG BL 69	Buckwheat seeds	*Lactobacillus* spp.
44	MIUG BL 32	Millet seeds	*Streptococcus* spp.
45	MIUG BL 19	Emmental cheese	*Leuconostoc* spp.
48	MIUG BL 40	Sesame seeds	

* Microorganisms Collection of Dunărea de Jos University of Galați (acronym: MIUG), Galati, Romania.

**Table 2:** Production of exopolysaccharides by presumptive LAB strains.

Strain No.	Absorbance ^1^ (λ = 490 nm)	Exopolysaccharides Biosynthesized ^2^ (mg/mL)
1	0.847 ± 0.12	11.307
13	0.934 ± 0.11	17.854
29	0.910 ± 0.51	16.092
33	0.939 ± 0.25	18.257
35	0.956 ± 0.08	19.516
36	1.004 ± 0.24	23.193
39	0.974 ± 0.25	21.027
45	0.933 ± 0.41	17.779
48	0.994 ± 0.36	22.387

^1^ Average values of three replicates ± standard deviation; ^2^ Results of the phenol-sulfuric acid method using glucose as standard (> 10 mg/mL).

**Table 3:** Antimicrobial properties of the flour extracts fermented by the presumptive LAB strains.

Strain No.	Chickpea Flour Extract (CFS)				Quinoa Flour Extract (CFS)				Buckwheat Flour Extract (CFS)			
	*A. niger* ^1^	*A. flavus* ^1^	*Penicillium* spp.^1^	*Bacillus* spp.^2^	*A. niger* ^1^	*A. flavus* ^1^	*Penicillium* spp.^1^	*Bacillus* spp.^2^	*A. niger* ^1^	*A. flavus* ^1^	*Penicillium* spp.^1^	*Bacillus* spp.^2^
1	++	−	−	+++	−	−	−	+++	−	−	−	++
2	−	−	−	+++	−	−	−	+++	−	−	−	++
13	+	+	−	++	−	−	−	++	−	−	−	++
16	−	+	−	+++	−	−	−	++	−	−	−	++
20	+	−	+	++	+++	−	−	+++	−	−	−	++
24	−	−	+	+++	+++	−	−	+++	+++	−	−	+++
29	−	−	+	++	+++	−	−	+++	+++	−	−	+++
30	−	−	−	+++	+++	−	−	+++	+++	−	−	++
31	−	−	−	+	+++	−	−	++	+++	−	−	++
33	−	−	−	+++	+++	−	−	+++	−	−	−	+++
35	−	−	−	++	−	−	−	++	−	−	−	++
36	−	−	−	+++	−	−	−	+++	−	−	−	++
38	−	+	−	++	++	++	−	+	−	−	−	++
39	−	+	−	++	−	−	−	+++	−	−	++	+++
41	−	−	+	++	−	−	−	++	+++	−	++	+++
43	−	−	++	+++	−	+	−	+++	+++	−	++	−
44	−	−	+	++	−	−	−	+++	−	−	++	+++
45	−	−	+	++	−	−	−	+++	−	−	+	++
48	−	+++	−	++	−	−	+	+++	+++	−	++	+++

Average values of three replicates.^1^ (–) no inhibition,(+) inhibition of spore formation,(++) inhibition of spore formation with a small zone of inhibition around the wells, (+++) good inhibition of the mycelium growth and sporulation; ^2^ (–) no inhibition, (+) 10–15 mm inhibition zone, (++) 16–21 mm inhibition zone, (+++) > 21 mm inhibition zone.

**Table 4:** Antifungal activity of fermented (**a**) chickpea, (**b**) quinoa, and (**c**) buckwheat extracts after undergoing thermal and acidification treatments. The fermentations were carried out throughout 72 h at 37 °C with *Lactobacillus* spp. no. 24 and *Leuconostoc* spp. no. 48.

Strain No.	Treatment	Chickpea Flour Extract (a)			Quinoa Flour Extract (b)			Buckwheat Flour Extract (c)		
		*A. niger*	*A. flavus*	*Penicillium* spp.	*A. niger*	*A. flavus*	*Penicillium* spp.	*A. niger*	*A. flavus*	*Penicillium* spp.
Control		26.00 ± 1.41	37.00 ± 0.00	24.75 ± 1.06	30.50 ± 0.71	36.00 ± 0.00	25.25 ± 1.06	28.50 ± 0.71	35.50 ± 0.71	27.00 ± 1.41
24	Untreated	30.00 ± 2.12	45.50 ± 0.71	23.5 ± 0.71	30.25 ± 3.18	52.25 ± 6.01	34.00 ± 0.00	34.00 ± 0.00	56.50 ± 12.02	30.00 ± 3.54
	I, %	n.d.	n.d.	5.05	0.82	n.d.	n.d.	n.d.	n.d.	n.d.
48	Untreated	31.25 ± 0.35	54.00 ± 3.54	21.75 ± 5.30	32.75 ± 0.35	54.25 ± 1.06	23.50 ± 8.49	32.75 ± 4.60	42.25 ± 0.00	34.75 ± 3.89
	I, %	n.d.	n.d.	12.12	n.d.	n.d.	6.93	n.d.	n.d.	n.d.
24	60 °C	27.50 ± 2.12	53.00 ± 2.12	31.25 ± 6.01	33.00 ± 1.41	43.75 ± 10.25	20,88 ± 0.53	32.00 ± 0.00	52.00 ± 3.54	35,75 ± 6.72
	I, %	n.d.	n.d.	n.d.	n.d.	n.d.	17.33	n.d.	n.d.	n.d.
48	60 °C	31.00 ± 0.00	47.50 ± 2.12	25.00 ± 1.41	33.25 ± 1.06	51.00 ± 4.24	22.08 ± 10.49	32.25 ± 2.47	53.25 ± 5.30	31.75 ± 10.25
	I, %	n.d.	n.d.	n.d.	n.d.	n.d.	12.54	n.d.	n.d.	n.d.
24	80 °C	36.08 ± 6.25	55.00 ± 0.00	38.50 ± 0.71	44.75 ± 3.89	52.50 ± 0.71	18.79 ± 5.96	44.50 ± 2.83	54.50 ± 0.00	24.33 ± 0.94
	I, %	n.d.	n.d.	n.d.	n.d.	n.d.	25.60	n.d.	n.d.	9.88
48	80 °C	41.00 ± 2.12	51.25 ± 1.77	22.00 ± 1.41	44.50 ± 2.12	52.25 ± 1.06	31.50 ± 10.61	34.88 ± 9.37	53.75 ± 0.35	19.05 ± 7.71
	I, %	n.d.	n.d.	11.11	n.d.	n.d.	n.d.	n.d.	n.d.	29.44
24	121 °C	42.75 ± 13.79	39.25 ± 7.42	26.53 ± 5.27	28.30 ± 7.70	41.00 ± 3.54	41.25 ± 3.18	35.00 ± 0.00	37.88 ± 9.37	36.50 ± 0.00
	I, %	n.d.	n.d.	n.d.	7.20	n.d.	n.d.	n.d.	n.d.	n.d.
48	121 °C	41.50 ± 0.00	43.50 ± 1.41	25.00 ± 0.00	40.00 ± 0.00	40.75 ± 6.72	55.00 ± 0.00	35.00 ± 0.00	45.50 ± 3.54	30.00 ± 0.00
	I, %	n.d.	n.d.	n.d.	n.d.	n.d.	n.d.	n.d.	n.d.	n.d.
24	pH 3.5	25.25 ± 1.77	46.75 ± 0.35	32.25 ± 10.25	29.75 ± 0.35	39.50 ± 2.12	36.00 ± 7.07	25.75 ± 0.35	42.75 ± 1.77	30.00 ± 0.00
	I, %	2.88	n.d.	n.d.	2.46	n.d.	n.d.	9.65	n.d.	n.d.
48	pH 3.5	27.50 ± 3.54	44.50 ± 0.71	25.00 ± 0.00	29.00 ± 0.71	46.00 ± 0.71	26.00 ± 0.00	24.75 ± 0.35	46.00 ± 0.71	30.00 ± 0.00
	I, %	n.d.	n.d.	n.d.	4.92	n.d.	n.d.	13.16	n.d.	n.d.
24	pH 5.5	20.75 ± 1.77	39.00 ± 0.00	36.25 ± 2.47	32.00 ± 2.83	37.50 ± 0.71	32.13 ± 11.14	28.75 ± 0.35	36.25 ± 0.35	34.50 ± 2.12
	I, %	20.19	n.d.	n.d.	n.d.	n.d.	n.d.	n.d.	n.d.	n.d.
48	pH 5.5	29.50 ± 9.90	40.25 ± 1.06	39.50 ± 0.00	38.75 ± 3.89	36.50 ± 2.12	38.00 ± 0.71	32.75 ± 1.77	36.75 ± 0.35	29.75 ± 15.91
	I, %	n.d.	n.d.	n.d.	n.d.	n.d.	n.d.	n.d.	n.d.	n.d.
24	pH 7.5	26.75 ± 3.89	38.75 ± 2.47	29.25 ± 0.35	30.00 ± 0.00	37.00 ± 0.00	25.00 ± 0.00	26.50 ± 2.12	35.50 ± 0.71	33.00 ± 2.12
	I, %	n.d.	n.d.	n.d.	1.64	n.d.	0.99	7.02	n.d.	n.d.
48	pH 7.5	26.00 ± 4.24	38.00 ± 2.12	30.50 ± 2.83	33.00 ± 0.00	37.50 ± 0.71	26.50 ± 2.12	25.50 ± 0.71	36.00 ± 1.41	34.00 ± 3.54
	I, %	n.d.	n.d.	n.d.	n.d.	n.d.	n.d.	10.53	n.d.	n.d.

The values are presented as average values of three (*n* = 3) replicates ± standard deviation for the mycelium growth (mm) used to determine the inhibition ratio, expressed as percent (equation 1); n.d. not determined.

**Table 5:** Antibacterial activity of the fermented flour extracts undergoing thermal and acidification treatments. The fermentations were carried out throughout 72 h at 37 ˚C with *Lactobacillus* spp. no. 24 and *Leuconostoc* spp. no. 48. The values are presented as average values of three (*n* = 3) replicates ± standard deviation.

Strain No.	Treatment	Chickpea Flour Extract	Quinoa Flour Extract	Buckwheat Flour Extract
24	Untreated	n.d.	20.00 ± 0.00	n.d.
48	Untreated	16.67 ± 2.31	16.00 ± 4.00	n.d.
24	60 °C	n.d.	17.33 ± 1.53	n.d.
48	60 °C	25.67 ± 1.15	28.67 ± 2.31	23.67 ± 1.53
24	80 °C	23.33 ± 1.53	24.00 ± 1.00	19.00 ± 1.73
48	80 °C	17.67 ± 1.15	20.33 ± 0.58	n.d.
24	121 °C	23.33 ± 2.89	26.67 ± 3.06	n.d.
48	121 °C	19.00 + 1.00	20.00 ± 1.73	n.d.
24	pH 3.5	22.33 ± 1.53	28.33 ± 2.89	25.33 ± 3.21
48	pH 3.5	n.d.	20.33 ± 0.58	n.d.
24	pH 5.5	23.67 ± 0.58	29.00 ± 1.73	21.67 ± 2.89
48	pH 5.5	21.67 ± 1.53	26.33 ± 2.31	n.d.
24	pH 7.5	n.d.	22.00 ± 1.73	n.d.
48	pH 7.5	n.d.	21.33 ± 2.31	n.d.

Antibacterial activity expressed as diameters of the zones of inhibition (mm); n.d. not determined.
